# Effects of Fuyou Formula on GnRH Secretion and Related Gene Expression in Treating Precocious Puberty

**DOI:** 10.3389/fphar.2022.852550

**Published:** 2022-03-11

**Authors:** Yi Zhang, Ning Sun, Meng Zhang, Qian Ding, Qian Wang, Yuguang Liang, Huan He, Yuxin Yang, Chunyan Guo

**Affiliations:** ^1^ Clinical Research Center, Beijing Children’s Hospital, Capital Medical University, National Center for Children’s Health, Beijing, China; ^2^ Department of Pharmacy, Beijing Children’s Hospital, Capital Medical University, National Center for Children’s Health, Beijing, China

**Keywords:** precocious puberty, fuyou formula, GnRH, gene expression, KISS1, ER

## Abstract

The Fuyou (Fy) formula is an in-hospital preparation consisting of traditional Chinese medicine (TCM) that has been used for treating precocious puberty (PP) for more than 20 years. In this study, we aimed to clarify the effect of the Fy formula and its major components on PP. To confirm the effect of the Fy formula on the release of hypothalamic gonadotropin-releasing hormone (GnRH), GT1-7 cells were treated with estrogen to build the model group and subsequently treated with the Fy formula and its major components to explore their effects on the secretion of GnRH. The level of GnRH in GT1-7 cells was determined using enzyme-linked immunosorbent assay. The results illustrated that, compared to the model group, the Fy formula inhibited the release of GnRH. In addition, the expression levels of proteins related to GnRH secretion, including GnRH, gonadotropin-releasing hormone receptor (GnRHR), Kiss-1 metastasis-suppressor (Kiss1), G-protein coupled receptor 54 (GPR54), estrogen receptor α (ERα), insulin-like growth factor-1 (IGF-1), and insulin-like growth factor-1 receptor (IGF-1R), were detected by real-time polymerase chain reaction (RT-qPCR). The results demonstrated that the Fy formula significantly reduced the level of GnRH secretion in the GT1-7 cell lines compared with the model group. Moreover, it significantly downregulated the expression of GnRH, GnRHR, Kiss1, GPR54, ERα, IGF-1, and IGF-1R. In summary, our results indicate that the Fy formula and its major components may inhibit the effects of estrogen, which alleviates PP through transcriptional regulation of target genes.

## Introduction

Precocious puberty (PP) is an endocrine disorder that is defined as puberty starting before the age of 9 in boys and eight in girls. Observational data from Europe show that breast development begins before the age of 8 years in 5% of girls. In China, the incidence of PP is approximately 1/50,000–10,000 population, and the male-to-female ratio is approximately 1:5–10 (Dong et al., 2019). The causes of pathological PP are normally categorized into central precocious puberty (CPP) and peripheral precocious puberty (PPP). Idiopathic CPP is the most common form of CPP, originating from the early activation of the hypothalamic-pituitary-gonadal (HPG) axis with pulsatile secretion of hypothalamic gonadotropin-releasing hormone (GnRH). Approximately 74% of girls with CPP have the idiopathic form (Bradley et al., 2020). CPP may also occur secondary to tumors involving the hypothalamus and congenital defects in neuronal migration, resulting in a heterotopic mass of GnRH-secreting neurons acting as an ectopic GnRH pulse generator. In the remaining situations, disruption of a normal inhibitory restraint on the onset of puberty is caused by an extensive variety of insults to the central nervous system (CNS) (Eugster and Pescovitz, 2001). These include hypothalamic tumors, cerebral malformations involving the hypothalamus, and congenital brain disorders, infections, or acquired injuries. PPP is often related to increased sex steroid levels independent of GnRH. It can be caused by virilizing tumors, including adrenal tumors, gonadal tumors, or human chorionic gonadotropin (hCG)-secreting germ cell tumors. Neoclassic tissues can then lead to an increase in androgen or estrogen production (Latronico et al., 2016). Familial male precocious puberty (FMPP) is caused by an activating mutation in the luteinizing hormone (LH) receptor gene. The activating mutation leads to the continuous activation of adenylate cyclase, resulting in gonadal autonomic hyperfunction. Congenital adrenal hyperplasia may lead to the excessive production of adrenal androgens. McCune-Albright syndrome and recurrent autonomous ovarian cysts caused by somatic activating mutations in the *GNAS* gene lead to an increase in the signal transduction of the GnRH signaling pathway. Children with PPP can easily develop CPP due to their early bone age and long-term hyperestrogenemia (Carel and Léger, 2008).

Puberty onset is thought to integrate diverse genetic and environmental signals (Leka-Emiri et al., 2017). The hypothalamic secretion of GnRH has been established as a pivotal pathway for initiating puberty onset. The synthesis and secretion of GnRH neurons in the hypothalamus are essential for the regulation of hormonal cascade effects, including pituitary gonadotropin release, ovarian maturation, and estrogen production. All of these hormonal events are necessary for normal sexual maturation and reproductive function. The release of GnRH activates the synthesis and secretion of LH and follicle-stimulating hormone (FSH) from the anterior pituitary, thus leading to the stimulation of gonadal function (Carel et al., 2004). Briefly, LH initiates the growth and ovulation of the corpus luteum in girls and release of androgen in boys. FSH mediates the formation and maturation of ovarian follicles in girls and spermatogenesis in boys, inducing secondary sexual characteristics (Kanasaki et al., 2017). Reproductive control of the HPG axis also facilitates negative gonadal feedback. GnRH is not the only hormone involved in puberty onset but is the most important factor identified to date. Therefore, the regulation of GnRH secretion and expression is critical for the pathogenesis of PP.

The pharmacological therapy for PP includes GnRH analogs (GnRHas), GnRH antagonists, and traditional Chinese medicine (TCM) Fy formula. GnRHas are the gold-standard management for CPP, as they provide continuous stimulation of pituitary gonadotrophs, resulting in the downregulation of the HPG axis and thus leading to decreased secretion of LH and FSH (Eugster and Pescovitz, 2001). Numerous studies have demonstrated that the use of GnRHas results in the stabilization of pubertal symptoms. Local side effects include pain at the injection site, sterile abscesses, and implant site reactions (Aguirre and Eugster, 2018). Other side effects include headache, hot flashes, decreased bone density, and vaginal bleeding (Fuqua, 2013). Several GnRHas have been synthesized and are currently under investigation in clinical trials. They exhibit high-affinity binding to the human GnRH receptor (GnRHR), leading to a rapid decrease in gonadal sex steroids to castrate levels; however, the detailed mechanism for this is still under investigation (M. Chen and Eugster, 2015).

TCM treating PP includes Zhibai Dihuang wan, Dabuyin wan and Fy formula. Zhibai Dihuang wan and Dabuyin wan were applied to yin deficiency, fire hyperactivity syndrome, phlegm dampness stagnation syndrome, liver depression and fire transformation syndrome. However, the recommendation level for the use of these two medicines are low, and there is no indication in the drug instruction. At present, there is no Chinese patent medicine with definite curative effect for treating PP are commercially available. The Fy formula is an in-hospital preparation used at Beijing Children’s Hospital, and it is composed of TCM and was developed by pediatric gynecologists according to the pathogenesis, etiology, and physical characteristics of children with PP. It was approved by the National Medical Products Administration (NMPA) as a compound preparation for TCM in hospitals in 2001. In the study of Fy formula treatment of 60 female with PP, it has been showed that the total effective rate is 83.3%. The changes of 60 cases before and after treatment were breast nucleus index, blood E_2_ level, number of positive cases of vaginal cell smear, bone age, uterine and follicular volume. The treatment can improve the symptoms of liver depression, yin deficiency and fire hyperactivity, reduce the level of estrogen and delay the speed of bone age maturation (Liu et al., 2009). It has also been reported that the Fy formula is able to regulate early pubertal symptoms, reducing the size of the mammary nucleus and effectively controlling estrogen levels and bone age. It has also been shown that the Fy formula exerts an inhibitory effect on female ovarian cysts complicated by PP, leading to a reduction in E_2_ levels and postponing the rate of bone maturation with no evident adverse effects (Pan et al., 2019).

It has been showed that the Fy formula induces downregulation of the mRNA expression of kiss1, GPR54, and GnRH in female rats (Bai et al., 2020). A previous integrated pharmacological study on the mechanism of Fy formula in treating PP demonstrated that it can effectively reduce the levels of FSH, LH, and E_2_ in Sprague-Dawley rats (Guo et al., 2021b). Also, the TCM-chemical component-target-pathway study based on integrated pharmacology illustrated that ERα, ERβ, IGF, and IGF1 are associated with PP, so these can be potential therapeutic targets for PP (Guo et al., 2021a). Therefore, in this study, we aimed to explore the effects of the Fy formula on the secretion of GnRH and expression of related genes in the treatment of PP.

## Materials and Methods

### Materials and Reagents

Dulbecco’s modified Eagle medium (DMEM) was purchased from Corning (NY, United States). Fetal bovine serum (FBS) was obtained from Gibco (Grand Island, NY). Dimethyl sulfoxide (DMSO) was purchased from Sigma-Aldrich (St. Louis, MO, United States). Penicillin-streptomycin and 0.25% trypsin-EDTA were purchased from MacGene (Beijing, China). *Mycoplasma* Prevention Reagent (MycAway™) was obtained from Yeasen Biotech (Hong Kong, China). Phosphate-buffered saline (PBS) was purchased from Solarbio (Beijing, China).

The TCM standards estradiol (E_2_, serial number: 100,182-21,906, purity: 96.3%), quercetin (serial number: 100081-201610, purity: 99.90%), and luteolin (serial number: 111520-202006, purity: 94.40%) were purchased from the National Institutes for Food and Drug Control (Beijing, China). Apigenin (serial number: B20981-20 mg, purity: 98.00%) was purchased from Shanghai Yuanye Biotechnology Co., Ltd. (Shanghai, China). The reagents were dissolved in DMSO (Sigma-Aldrich) and stored at 4°C.

### Fy Formula Preparation

The Fy formula is an in-hospital preparation obtained by mixing the following 12 herbs: *Prunella vulgarism* L (Xiakucao), *Carapax Trionycis* (Cubiejia), *Gentiana scabra* Bunge (Longdan), *Chrysanthemum morifolium* (Ramat.) Hemsl (Juhua), *Lycium chinense* Mill (Digupi), *Alisma plantago-aquatica* L (Zexie), *Scrophularia ningpoensis* Hemsl (Xuanshen), *Paeonia suffruticosa Andrews* (Mudanpi), *Rehmannia glutinosa* (Gaertn.) DC (Shengdihunag), *Hordeum vulgare* L (Maiya), *Concha oetreae* (Muli), and *Thallus laminariae* (Kunbu) at the ratio of 1.5:1:0.6:0.6:1:1:1.5:0.6:1.2:2:3:1. All herbs were purchased from the Beijing Bencao Fangyuan Pharmaceutical Group Co. Ltd., and the Fy formula was prepared by the Preparation Center of Beijing Children’s Hospital (approval number: Z20053679; lot number: 20201202).

### Cell Cultures

The GT1-7 cell line (mouse GnRH neuronal cell line) was kindly provided by Prof. P. Mellon (University of California, San Diego, CA, United States). GT1-7 cells were grown in a monolayer culture in DMEM (Corning) supplemented with 10% FBS (Gibco), 1% penicillin-streptomycin (Macgene), and 0.5% *Mycoplasma* Prevention Reagent (Yeasen Biotech). The cultures were incubated at 37°C in an atmosphere of 5% CO_2_ in a 25 mm flask (Corning) for 2 days after seeding, with a medium change at 24 h. The cells were then washed twice with PBS and digested with 0.25% trypsin-EDTA (MacGene). When more than half of the cells were observed to become round under a microscope, serum-containing medium was added to terminate the digestion. After obtaining a single-cell suspension, the cells were cultured in an incubator and inoculated three times for subsequent experiments. The cells were treated with different treatments in serum-free medium (SFM) for 24 h depending on the experiment.

### CCK-8 Assay

Cell counting kit-8 (CCK-8) was purchased from Solarbio (Beijing, China). To assay the toxicity of the Fy formula, quercetin, luteolin, apigenin, and GT1-7 cells were seeded in 96-well plates at a density of 2.0×10^5^ cells/well. After 24 h of incubation, the cells were pretreated with 100 pmol/L E_2_ in SFM overnight. The cells were then incubated with 75, 150, 225, 300, 450, and 525 μg/ml of Fy formula or 5, 10, 15, 20, 30, and 40 μmol/ml of quercetin, luteolin, and apigenin separately in SFM for 24 h. Subsequently, the cells were treated with 10 μL of CCK-8 solution (Gibco) for 1 h at 37°C and 5% CO_2_. The optical density (OD) was determined by measuring the absorbance at 450 nm using a microplate reader (BioTek Synergy, United States).

### ELISA

To determine the concentration of GnRH, GT1-7 cells were seeded in 24-well plates. The cells were treated with E_2_, Fy, quercetin, luteolin, and apigenin, as previously described. After treatment, the supernatants were collected. Relative GnRH concentrations in the supernatant were determined using the mouse GnRH ELISA kit from mlbio (Shanghai, China) with serial dilutions of 80, 40, 20, 10, 5, and 0 mIU/mL as a standard curve.

### RNA Extraction and Quantitative Real-Time Polymerase Chain Reaction

The cells were seeded in 6-well plates and treated with E_2_, Fy, and TCM, as described previously. At the end of the treatment, total RNA was extracted from GT1-7 cells using an RNA Easy Fast Tissue/Cell Kit (TIANGEN BIOTECH Co., Ltd, Beijing, China) according to the manufacturer’s protocol. The RNA samples were quantified using a microplate reader (BioTek Synergy, United States) at 260/280 nm. First-strand cDNA was prepared using 2 μg RNA reverse-transcribed with a FastKing RT Kit (TIANGEN BIOTECH Co., Ltd, Beijing, China). The synthesized first-strand cDNA was stored at −80°C until use. mRNA expression was analyzed using a 7500 Fast Real-Time PCR system (Applied Biosystems, United States) with the following thermocycling conditions: 50°C for 2 min, 95°C for 10 min, and 40 cycles of 95°C for 15 s and 60°C for 60 s. Single-stranded oligonucleotide primer sets were designed (Tianyi Huiyuan, Beijing, China) to target *β-actin, Erα, Kiss1, GPR45, GnRH, GnRHR, IGF1, and IGF1R*. The primer sequences used for qRT-PCR are listed in Table 1. Data were analyzed using the 2^ΔΔCt^ method, and mRNA expression was normalized to that of *β-actin.*

**TABLE 1:** Primer sequences used in quantitative real-time polymerase chain reaction.

Gene	Forward primer (5′—3′)	Reverse primer (3′—5′)
*β-actin*	ACT​CTT​CCA​GCC​TTC​CTT​C	ATC​TCC​TTC​TGC​ATC​CTG​TC
*GnRH*	GGG​AAG​ACA​TCA​GTG​TCC​CAG	CTC​GAG​CTT​CCG​TTG​GTA​GG
*GnRHR*	TGC​AGG​ACC​ACA​GAA​CTA​CAG	GTC​CAG​CAG​ACG​ACA​AAG​GA
*Kiss1*	GAT​GTC​TGC​AGC​CTG​AGT​CCC	AGG​CAT​TAA​CGA​GTT​CCT​GGG
*GPR54*	CTG​TCA​GCC​TCA​GCA​TCT​GG	AGC​AGC​GGC​AGC​AGA​TAT​AG
*ERα*	AAG​ACG​CTC​TTG​AAC​CAG​CA	CGA​GTT​ACA​GAC​TGG​CTC​CC
*IGF-1*	AAG​GCA​GTT​TAC​CCA​GGC​TC	GGC​CGA​GGT​GAA​CAC​AAA​AC
*IGF-1R*	TAC​CAG​CAT​TAA​CTC​CGC​TG	GCT​CGC​CTC​TCT​CGA​GTT​C

*GnRH*, gonadotropin-releasing hormone; *GnRHR*, gonadotropin-releasing hormone receptor; *Kiss1*, Kiss-1 metastasis-suppressor; *GPR54*, G-protein coupled receptor 54; *ERα*, estrogen receptor α; *IGF-1*, insulin-like growth factor-1; *IGF-1R*, insulin-like growth factor-1 receptor.

### Statistical Analysis

All experiments were performed in triplicate independently and data were expressed as mean ± SD. Significant differences were analyzed by one-way analysis of variance (ANOVA), and the data were plotted using Prism7 software (GraphPad software, Inc., San Diego, United States). Statistical significance was determined using *p* < 0.05.

## Results

### Composition Analysis of the Fy Formula

The TCM components of the Fy formula are listed in Table 2. *Prunella vulgaris* L. and *Carapax trionycis* exert effects on the liver that can nourish yin, moderate heat, and relieve congestion. *Gentiana scabra* Bunge, *Chrysanthemum morifolium* (Ramat.) Hemsl, *Lycium chinense* Mill, *Alisma plantago-aquatica* L., *Scroophularia ningpoensis* Hemsl, *Paeonia suffruticosa Andrews*, and *Rehmannia glutinosa* (Gaertn.) DCs can nourish yin, eliminate dampness, and cool blood. *Hordeum vulgare* L, *Concha oetreae* and *Thallus laminariae* act on the liver to relieve congestion and are used as adjuvants. As previously reported, five compounds were recognized with the HPLC-MS/MS method from Fy formula including Luteolin, Quercetin, Apigenin, Kaempferol and Emodin. Also, the concentration of these five target components in Fy Formula were determined using preliminary LC-MS/MS method, the concentration of Luteolin, Quercetin and Apigenin are much higher than Kaempferol and Emodin in Fy formula. Therefore, the compounds with higher concentration were selected in the experiment. (Guo et al., 2021a). We aimed to determine the effects of the Fy formula and its major components, including luteolin, quercetin, and apigenin, on GnRH secretion and related gene expression in PP treatment.

**TABLE 2:** Composition of the Fy formula.

Chinese name	Scientific name	Family	Lot no	Place of origin	Parts of plant used
Xia Ku Cao	*Prunella vulgaris* L	*Lamiaeae*	20201010	Jiangsu, China	Driod orial parts
Cu Bie Jia	*Carapax Trionycis*	*Trionyxsinensis Wiegmann*	20201018	Hubei, China	Carapace
Long Dan	*Gentiana scabra* Bunge	*Gentianaceae*	20200927	Yunnan, China	Dried roots and rhizomes
Ju Hua	*Chrysanthemum morifolium* (Ramat.) Hemsl	*Compositae*	20201027	Anhui, China	Capitulum
Di Gu Pi	*Lycium chinense* Mill	*Solanaceae*	20201105	Hebei, China	Dried root bark
Ze Xie	*Alisma plantago-aquatica* L	*Alismataceae*	20201126	Fujian, China	Dried tuber
Xuan Shen	*Scroophularia ningpoensis* Hemsl	*Scrophulariaceae*	20201019	Zhejiang, China	Dried root tuber
Mu Dan Pi	*Paeonia suffruticosa Andrews*	*Paeoniaceae*	20201123	Anhui, China	Dried root bark
Sheng Di Huang	*Rehmannia glutinosa* (Gaertn.) DC	*Plantaginaceae*	20201104	Henan, China	Dried root tuber
Mai Ya	*Hordeum vulgare* L	*Triticum*	20201030	Hebei, China	Dried ripe fruit
Mu Li	*Concha oetreae*	*Ostrea*	20200917	Guangdong, China	Shell
Kun Bu	*Thalluslaminariae*	*Laminaria*	20200922	Fujian, China	Dried lobes

### Effects of Luteolin, Apigenin, Quercetin, and the Fy Formula on the Proliferation of GT1-7 Cells

**FIGURE 1:**
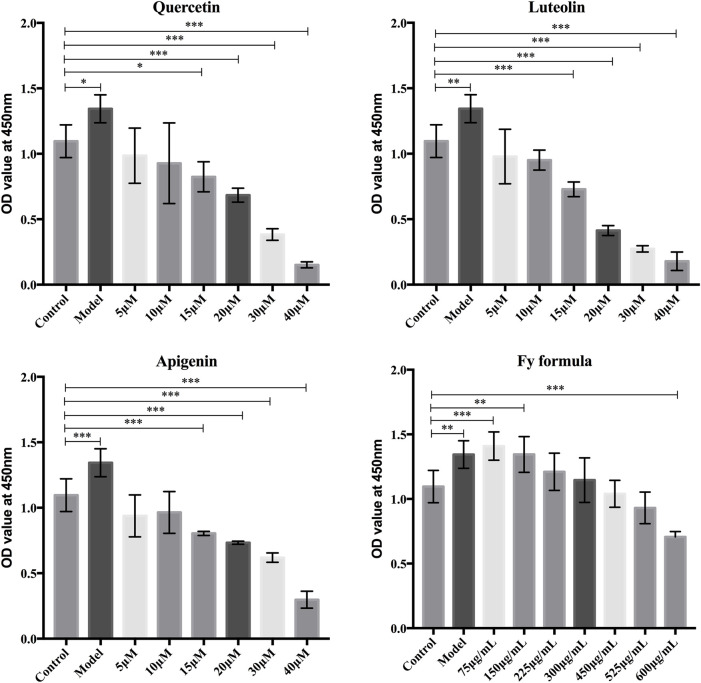
Effects of luteolin, apigenin, quercetin, and the Fy formula, at different concentrations, on GT1-7 cells. The CCK-8 assay was conducted to determine cell proliferation in the GT1-7 cells after treatment with luteolin, apigenin, quercetin, and the Fy formula at different concentrations.

The nontoxic concentrations of Fy and its major chemical components in GT1-7 cells were evaluated based on cell viability. The CCK-8 assay was performed to examine the proliferation of GT1-7 cells following treatment with different concentrations of luteolin, apigenin, quercetin, and Fy. As demonstrated in Figure 1, the GT1-7 cells treated with high concentrations of the Fy formula and its major components showed reduced cell proliferation activity compared to the control group cells. The cell proliferation activity of the GT1-7 cells was not significantly different at concentrations of 5 and 10 μM for luteolin, apigenin, and quercetin and concentrations of 450 μg/ml and 525 μg/ml for the Fy formula compared to the control group. To ensure that the Fy formula was administered at a concentration sufficient to exert the desired effect, the final concentrations of 10 μM and 525 μg/ml were selected for use in subsequent experiments.

### The Fy Formula, Luteolin, Apigenin, and Quercetin Inhibit GnRH Secretion in GT1-7 Cells

**FIGURE 2:**
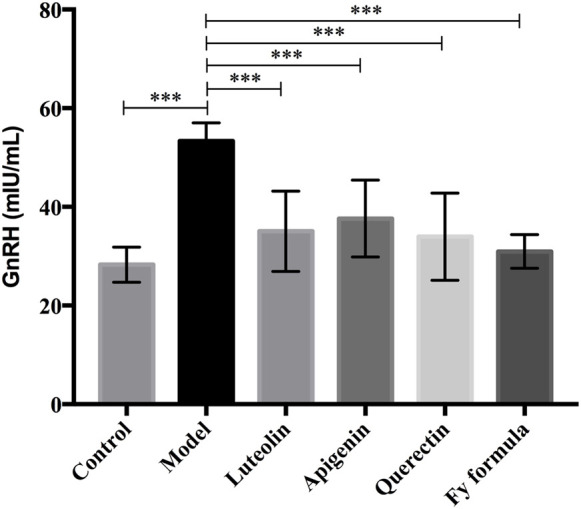
Effects of luteolin, apigenin, quercetin, and the Fy formula on GnRH secretion. Cells pretreated with E_2_ stimulate the secretion of GnRH, and the level of GnRH secretion decreases when coincubated with luteolin, apigenin, quercetin, and the Fy formula. ***p < 0.0001 compared to the model group.

After pretreatment of the GT1-7 cells with E_2_, ELISA was performed to determine the level of GnRH in the GT1-7 cells. The GnRH concentration in the culture medium is shown in Figure 2. Comparing the model and control groups, the E_2_ treatment resulted in an increase in GnRH secretion. Moreover, treatment with the Fy formula, luteolin, apigenin, and quercetin led to a significant reduction in the concentration of GnRH in the culture medium and thus inhibited the increased level of GnRH secretion caused by E_2_ in GT1-7 cells.

### The Fy Formula, Luteolin, Apigenin, and Quercetin Inhibit *GnRH, GnRHR, Kiss1, GPR54, ERα*, *IGF-1,* and *IGF-1R* Expression in GT1-7 Cells

**FIGURE 3:**
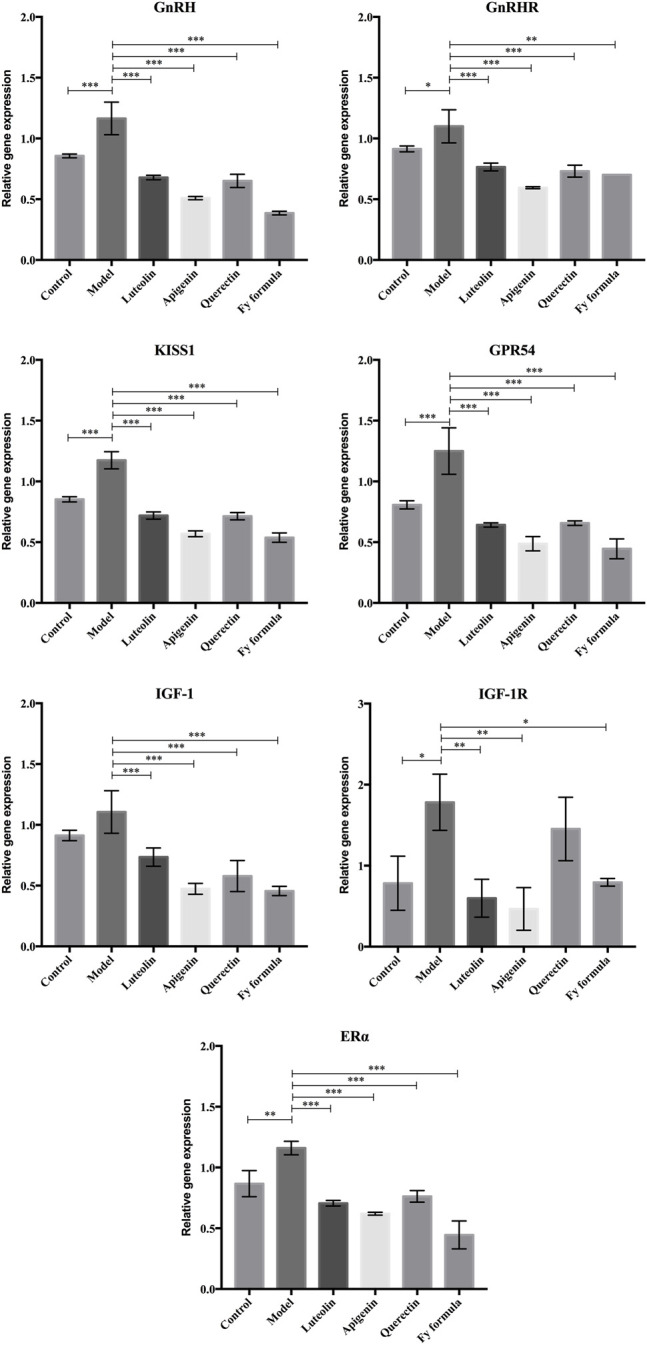
Effects of luteolin, apigenin, quercetin, and the Fy formula on the expression of *GnRH, GnRHRc, Kiss1, GPR54, ERα IGF-1*, and *IGF-1R*, which are involved in GnRH secretion. *p < 0.05, **p < 0.001, and ***p < 0.0001 compared to the model group.

To further analyze whether the potential molecular mechanism of the Fy formula on GnRH secretion in GT1-7 cells is via the GnRH receptor, E_2_ receptor, Kiss1/GPR54 signaling pathway, or IGF-1, the mRNA expression levels of these genes were quantified by RT-qPCR (Figure 3). GT1-7 cells treated with E_2_ showed significantly upregulated gene expression of *GnRH, GnRHR*, *Kiss1*, *GPR54*, *ERα*, *IGF-1,* and *IGF-1R*. In contrast, GT1-7 cells treated with the Fy formula, luteolin, apigenin, and quercetin showed downregulated expression of all genes involved in GnRH secretion. These results indicate that the effect of the Fy formula is mediated by inhibiting the expression of *GnRH* itself, as well as *GnRHR, Kiss1, GPR54, ERα, IGF-1,* and *IGF-1R*, which are related to GnRH secretion.

## Discussion

In our previous study, five major chemical components of the Fy formula were identified using HPLC-MS/MS: luteolin, quercetin, apigenin, kaempferol, and emodin (Guo et al., 2021b). Three compounds with higher concentration were selected in the experiment including Luteolin, Quercetin and Apigenin. The association of ERα, ERβ, IGF, and IGF1 with PP are identified by “TCM-chemical component-target-pathway” study based on integrated pharmacology, which indicated that these proteins can be the potential targets for treating PP (Guo et al., 2021b). Moreover, it has been reported that the treatment with Fy formula can result in a significantly reduction in the level of E2, LH and FSH in Sprague-Dawley rats (Guo et al., 2021b). Furthermore, it has been illustrated that the Fy formula is able to downregulate the expression of Kiss1, GPR54, and GnRH in female rats (Bai et al., 2020).

As an in-hospital preparation at Beijing Children’s Hospital, the Fy formula has been used for the treatment of PP for more than 20 years. Clinical data illustrated that the Fy formula significantly reduced the level of estrogen in the blood serum of patients. It can also delay bone maturation and decrease the mammary gland size in women with PP. Furthermore, TCM research has revealed that the herbs used in the preparation of the Fy formula have intervention effects on ovarian cysts in girls complicated with PP. Taken together, these findings indicate the clinical benefits of the Fy formula, which is an advantageous therapeutic approach owing to its low cost. However, the mechanism of action of the Fy formula in treating PP has not been fully clarified.

GT1-7 cells are a valuable GnRH-expressing cell model with a number of characteristics common to normal GnRH neurons. In addition, GT1-7 cells express a number of genes relevant to reproduction, circadian rhythm, and energy homeostasis, including *GnRH*, *Kiss1*, and *GPR54*. Estrogen has been proven to be the main regulator of GnRH neuronal function in the female brain, which possesses a bimodal effect on the hypothalamic–pituitary axis. In GT1-7 cells, estrogen exerts a stimulatory effect at low concentrations (Qian et al., 2020) and an inhibitory effect at high concentration on the secretion of GnRH and gonadotropin (Roy et al., 1999). It has been suggested that the binding sites for E_2_ in the plasma membrane of GT1-7 cells share structural homology with classical estrogen receptors (ERs) at their carboxy-terminal domain (Morales et al., 2007). To investigate the effects of the Fy formula on GnRH secretion and related gene expression in PP treatment, we incubated GT1-7 cells with E_2_ (100 pmol/L) for 24 h, followed by the Fy formula, luteolin, apigenin, or quercetin for another 24 h. Our results showed that E_2_ treatment increased the release of GnRH from the GT1-7 cells and established a PP model in GT1-7 cells. However, the level of GnRH in the GT1-7 cells was decreased by treatment with the Fy formula, luteolin, apigenin, and quercetin compared to that in the model group. This suggests that the Fy formula can significantly reduce GnRH secretion.

To determine the role of potential targets associated with GnRH secretion in the effects of the Fy formula on PP, gene expression was analyzed. Our results showed that *GnRHR, Kiss1, GPR54, ERα*, *IGF-1,* and *IGF-1R* mRNA levels were higher in the model group than in the control group. We also found that, compared to the model group, the Fy formula significantly downregulated the expression of GnRH secretion-related genes, including *GnRHR, Kiss1, GPR54, ERα*, *IGF-1,* and *IGF-1R*, in GT1-7 cells. Taken together, these results indicate that the Fy formula and its major components, including luteolin, apigenin, and quercetin, are able to inhibit GnRH secretion in GT1-7 cells by inhibiting the expression of related genes, weakening the binding between signaling molecules and their receptors, and ultimately reducing the pulsed secretion of GnRH, thus reducing the activation of downstream pituitary and gonadal development and alleviating the symptoms of PP.

GnRH is a decapeptide that serves as a vital element in the regulation of the reproductive cycle and sexual maturation. GnRH drives the release of pituitary gonadotropic hormones, including LH, FSH, and gonadotropin, by interacting with pituitary gonadotropes through binding to its high-affinity receptor GnRHR on the cell surface (Tzoupis et al., 2020). GnRHR belongs to the G protein-coupled receptor family (GPCRs) that is characterized by seven transmembrane domains. It has been demonstrated that GnRHR gene expression is dependent on GnRH pulse in rat pituitary cultures, with increased mRNA expression levels being observed under high pulse frequency. In response to varying GnRH pulses, GnRHR appears to differentially activate multiple distinct signaling pathways implicated in the synthesis of both LH and FSH (Stamatiades and Kaiser, 2018). In our study, it was shown that the Fy formula and its major components could significantly suppress the expression of both GnRH and GnRHR, indicating that the Fy formula may delay pituitary gonadotropic hormone release and alleviate PP.

Recent research has demonstrated that signaling by kisspeptin through its receptor, G protein-coupled receptor GPR54 (also called Kiss1R), is the most potent stimulator of GnRH-induced gonadotropin release (Chan et al., 2009 and; Blaustein, 2010). Kisspeptin is encoded by the *KISS1* gene, which contacts GnRH neurons within the hypothalamus and induces GnRH release by binding to its receptor, GPR54 (Mayer and Boehm, 2011). Subsequently, GnRH reaches the pituitary gland through portal circulation and initiates the secretion of pituitary gonadotropins (Trevisan et al., 2018). Kisspeptin treatment in immature rodents and primates was able to induce activation of the gonadotropic axis and precocious pubertal development (Kanasaki et al., 2017). Serum kisspeptin-54 levels were higher in girls with CPP than in prepubescent controls, implying that kisspeptin secretion may stimulate the onset of puberty (C.-Y. Chen et al., 2013). It has also been reported that in GPR54-overexpressing GT1-7 cells, intracellular signaling, such as extracellular signal-regulated kinase (ERK) activation and protein kinase A (PKA) signaling pathways, were activated, resulting in increased GnRH receptor expression in response to kisspeptin (Kanasaki et al., 2017 and; Kang et al., 2009). Therefore, the development of kisspeptin antagonists may be a new approach for treating PP. In our results, we found that the Fy formula can target kisspeptin and its receptor GPR54 by suppressing their mRNA expression, thus inhibiting their activity.

IGF-1 is an important somatotropic hormone that mediates the regulation of the reproductive axis. In addition, it has emerged as a prime candidate for having a significant role in the onset of puberty. IGF-1 may promote the secretion of prepubertal GnRH, and the level of IGF-1 increases in the circulation as puberty approaches, which can advance the timing of puberty (Dees et al., 2021). Multiple findings suggested that IGF-1 may prime pituitary gonadotrophs and stimulate the synthesis of GnRHR and FSH during puberty onset in prepubertal salmon. Also, IGF-1 enhances pituitary gonadotropic hormone release, which accelerates puberty onset in rats (Luckenbach et al., 2010). More recent findings have depicted a later action of IGF-1 in regulating the synthesis and release of kisspeptin. It has been reported that IGF-1 activates kiss-1 in female rats, expressing kisspeptins that are involved in the secretion of pituitary gonadotropins at puberty, as previously described (Hiney et al., 2009). Furthermore, girls with CPP have remarkably higher levels of IGF-1 and insulin than healthy girls (Sørensen et al., 2012 and; Kanety et al., 1996). Our research on GT1-7 cell lines further corroborated that the Fy formula appears to downregulate the expression of IGF-1 and its receptor, inhibiting their activity in pubertal development and hence relieving PP.

With respect to the E_2_, several studies have reported that it alters pulsatile GnRH secretion through the binding and activation of ERs (Thomas and Dong, 2006). The ER is a member of the nuclear receptor superfamily that participates in the transcriptional regulation of multiple genes. The classic ER signaling pathway is initiated by the binding of estrogen to its receptor. Two isoforms of ER have been described: ERα and ERβ. This leads to receptor dimerization and subsequent combination with the estrogen response element located on the promoter of target genes, which finally activates gene transcription (Radovick et al., 2012). ERα may contribute to the feedback regulation of kisspeptin expression during pubertal development (Clarkson, 2013), which is associated with the restraint of GnRH release prior to puberty onset, followed by enhanced initiation of GnRH secretion and thus prompt reproductive maturation throughout puberty (Mayer et al., 2010). In contrast, ERβ may directly participate in estrogen regulation by regulating neuronal activity, gene expression, and pulsatile secretion of GnRH (Wolfe and Wu, 2012 and; Fixemer et al., 2003). Our research suggests that the Fy formula can inhibit this effect, demonstrating that the gene expression levels of both ERα and kiss-1 are repressed by the TCM components of the Fy formula.

However, this study remains few limitations that need to be considered. First of all, the effects of Fy formula against PP have been identified *in vitro*, the effects of Fy formula in treating PP *in vivo* still need to be verified in the future by comparing the level of GnRH secretion and gene expression before and after the treatment of Fy formula. Secondly, the mechanism of protein interactions in treating PP still need to be further clarified.

## Conclusion

In conclusion, we have shown evidence of inhibitory effect of Fy formula in GnRH secretion in GT1-7 cell lines and have shown that Fy formula down regulates GnRH gene expression *in vitro*. The Fy formula also suppresses the expression level in all genes that involved in the GnRH secretion including GnRHR, Kiss1, GPR54, ERα, IGF-1, and IGF-1R, and hence delay the pituitary gonadotropic hormone release and alleviating the symptoms of PP.

## Data Availability

The original contributions presented in the study are included in the article/Supplementary Material, further inquiries can be directed to the corresponding author.
